# Factors Influencing the Data Communication Equipment Competence in Korean Older People

**DOI:** 10.3390/geriatrics7030070

**Published:** 2022-06-17

**Authors:** HeeYoung Kim

**Affiliations:** Department of Occupational Therapy, Honam University, Gwangju 62399, Korea; guruma2000@naver.com; Tel.: +82-062-940-5544

**Keywords:** aging, data communication equipment competence, older people

## Abstract

The purpose of this study was to identify the most vulnerable group among older Korean adults regarding information literacy. Once that was identified, the study aimed to provide basic data for developing strategies to improve information literacy by investigating the factors that influence the ability to utilize the Data Communication Equipment (DCE). The subjects included 10,073 older adults from the 10,299 participants of the 2017 Korean National Survey of Older Adults. The mean age of the older people was 74.06 years from a range of 65 to 106 years old. This study excluded the 216 individuals who did not complete the survey. The data were analyzed using the SPSS 18.0 program. A univariate analysis was performed to identify the most vulnerable group with regard to DCE competence. To investigate the factors that influence DCE competence, a logistic regression analysis was performed on the significant variables in the univariate analysis, while the nominal variables were treated as dummies. Senior citizens in Korea were less able to utilize DCE when they had higher ages, lower education levels, were women, lived alone, lower incomes, decreased sensory function, decreased cognitive function, negative value of learning, no lifelong learning, and smaller social networks. The factors influencing DCE competence in older adults were as follows: age, education level, income level, health status, cognitive function, social networks, lifelong learning, and the value of learning. For DCE competence in older adults to be effectively improved, adequate support must be provided to the vulnerable group. Furthermore, support for personalized DCE utilization seems essential and should consider a person’s age, education level, income, health status, cognitive function, social networks, lifelong learning and the value of learning.

## 1. Background

Digitization has influenced, and with concurrent rapid technological development, continues to change how people engage in occupations [1]. The global COVID-19 pandemic has continuously accelerated the use of digital devices since 2020. This includes personal computers and mobile phones as they allow daily activities such as online classes, online group meetings, and video chats without spatial constraint [2]. The national rate of Internet usage in South Korea was 91.8% in 2019, with 96.5% of the citizens aged ≥6 years owning a mobile phone, and a smart phone supply rate of 91.7% [3]. Digitization can provide possibilities for citizens to achieve good and equitable health and welfare, as well as independence and participation in community living [4].

Meanwhile, the older people have found it difficult to adapt to such digitization. According to the report on the digital divide by the Ministry of Science and ICT [5], the older adults in South Korea have shown a lower level of information literacy than known minority groups. These groups include the disabled, low-income households, agricultural, fishery communities, North Korean refugees, and marriage immigrants [6]. In fact, compared to the entire population, the level of digital information literacy in activities of daily living, such as transportation, map access, product purchase, and financial trade was low in older adults and caused difficulties in their daily living [7]. South Korea is facing growing longevity of the population and consequently, an increase in the number of single households containing older adults. Therefore, it is essential that older adults are able to obtain useful information for an independent lifestyle [8]. For the older people, the ability to search for and use information enhances their quality of life and helps in allowing more convenient living as the individuals become more productive [9]. Currently, the lack of necessary knowledge and techniques regarding this information era is likely to drive the older population into inequality due to information alienation.

The percentage of Korean adults aged ≥60 years who have a smart phone showed a steep increase from 19% in 2016 [10] to 76.6% in 2020 [5]. This means that the gap in physical access to digital use that is experienced by the older people is largely being resolved. However, information literacy does not simply depend on the possession of Data Communication Equipment (DCE), but the level of personal utilization of DCE leads to a different type of information gap [11]. It is important to examine competence regarding the utilization of DCE. Thus, DCE competence is the personal capacity for the practical use of information through the use of a DCE and the motivation to adapt to the digital era [12].

According to previous studies that were conducted on DCE competence, information literacy decreases in the Old-Old population (aged ≥85 years) [13], while it is higher for males than females [13,14]. A high level of education was correlated with a high level of interest in Internet access and digitization [9,13]. Meanwhile, poor health status and restricted daily activities were correlated with low information literacy [13]. Internet access was obstructed due to a lack of understanding of technical terms or a problem in physical functions, such as visual, auditory, sensory functions, aging, and muscular weakness [15]. Cognitive impairment or physical discomfort in the eye, neck, or waist can prevent digital access [16]. Some studies reported a positive correlation between income and Internet usage, awareness, and motivation [8,14]. However, another study in the field found no correlation between the two [13].

As most previous studies relied on a small sample size and a specific survey area, the results could not be generalized. Each study investigated a limited set of factors; an extensive analysis of all factors related to information literacy has not yet been performed.

Therefore, in this study, the factors related to DCE competence in older adults were analyzed using the data from the Korean National Survey of Older Adults. This allowed for a more extensive analysis with the aim to provide basic data for developing the strategies to improve information literacy in older adults, especially the vulnerable group that were identified based on the data.

## 2. Methods

### 2.1. Participants

In this study, a secondary data analysis was conducted using the data of the 2017 Korean National Survey of Older Adults. This study investigated the factors related to older Korean adults, including the household type, family relations, income, health status and functions, economic status, social activities in leisure, and living environment. The 2017 Korean National Survey of Older Adults was approved by Korea Statistics (Approval No. 11771). The 2017 Survey on Seniors applied for IRB to the ‘Bioethics Committee of Korea Institute for Health and Social Affairs’ and received IRB approval (No. 2017-11, 17-034-00) after screening [17]. For sampling, the raw data were stratified in two stages: first, for 17 cities and provinces; second, for nine provinces and Sejong City (excluding seven autonomous or metropolitan cities). As a result, approximately 10,000 individuals were used as an adequate sample size. Based on the number of older adults in the 2015 Population and Housing Census for each city and province, a root proportional allocation was performed for the two-stage stratification in cluster sampling. This survey was conducted between 12 June 2017 and 28 August 2018 by 60 professional researchers and comprised 15 teams consisting of 4 investigators and 1 instructor, all of whom were trained by the researchers in advance, based on the survey table that was designed by the researchers. The survey was conducted using a direct interview method for the older people aged 65 or older living in the household by visiting all households in the pre-sampled survey area in person. In other words, the survey subjects were older people aged 65 or older living in households, and 10 people in urban areas and 20 people in rural areas were completed for each survey district. In addition, for the calculation of statistics by city and province, more than 400 older people in each city and province were surveyed. The final sample set contained 10,073 individuals (excluding 216 incomplete responses) among older adults (≥aged 65 years) who had completed the survey (personal responses only).

### 2.2. Measure

#### 2.2.1. General Characteristics

The following factors related to older adults were analyzed: age, gender, income, marital status, cohabiting children, non-cohabiting children. Age was categorized into young-old (aged 65–74 years); mid-old (aged 75–84 years); and old-old (85– years). The mean age of the older adult was 74.06 years and it ranged from 65 to 106 years old. Sex was divided into male and female. Income, as an average monthly earning, was categorized into <0.3 million; <0.5 million and ≥0.3 million; <1 million and ≥0.5 million; <1.5 million and ≥1 million; <2 million and ≥1.5 million; and ≥2 million KRW. For marital status the responses were recorded as Yes or No. Education was categorized into Illiteracy, None, Elementary school, Middle school, High school, and University. 

#### 2.2.2. Self-Rated Health

To measure self-rated health, question B1 in the Korean National Survey of Older Adults was used. The data of the responses to the question ‘How would you rate your general health status?’ (as scored by 1 for Very healthy, 2 for Fairly healthy, 3 for Moderate, 4 for Fairly unhealthy, and 5 for Very unhealthy) were used without modification.

#### 2.2.3. Sensory Function

Visual function: To measure the visual function, the responses, No discomfort, Slight discomfort, and Much discomfort, regarding any discomfort in daily activities were used.

Auditory function: To measure the auditory function, the responses, No discomfort, Slight discomfort, and Much discomfort, regarding any discomfort in daily activities were used.

#### 2.2.4. Cognitive Function

The cognitive function was measured using the Mini-Mental State Examination-Korean version (MMSE-K) as developed by Folstein & McHugh [18] and translated and standardized by Kwon & Park [19]. The tool consisted of 12 questions with a total score of 30, where scores ≤19 confirmed dementia, scores 20–22 indicated suspected dementia, and scores ≥23 indicated normal. The inter-rater reliability of the tool is 0.99.

#### 2.2.5. Education Related Factors

The value of learning: The value of learning was measured based on the responses, Very high, Fairly high, Moderate, Fairly low, and Very low, to the question ‘How would you value your learning something new?’.

The experience of lifelong learning: For lifelong learning, the number of responses of Yes or No regarding current participation in lifelong learning was used.

#### 2.2.6. Social Networks

For social networks, the number of frequently visiting siblings and acquaintances was analyzed based on the response to the question ‘How many close relations including brothers and sisters, and neighbors and acquaintances do you have?’

#### 2.2.7. Data Communication Equipment (DCE) Competence

To measure the subject’s DCE competence, a questionnaire survey was conducted on the ability to utilize DCE (mobile phone, computer, tablet PC, Internet, TV, etc.). The questions regarded sending and receiving text messages; searching for information, including news and weather; taking photos and videos; listening to music (MP3, etc.); playing games; watching videos (movies, TV programs, etc.); using the SNS (Band, Kakao-talk, Twitter, Facebook, Instagram, Telegram, etc.); and online shopping. The response to each question was scored by 1 for yes and 0 for no. The total score was taken as the score of DCE competence. The construct validity of the ten questions in this survey was high (0.919) in the KMO test to determine the sampling adequacy for an exploratory factor analysis [17]. The fit of the model in Bartlett’s test of sphericity was 0.0001 significance probability (5% level of significance) [17]. For the factor analysis, the principal component analysis was used. An exploratory factor analysis was performed through Varimax, an orthogonal rotation method [17]. The result of the factor analysis showed that the correlation coefficient of each question was between 0.34 and 0.81 at maximum [17]. For commonality, the only item with a score <0.4 (0.339) was online shopping which was nevertheless included in the analysis as it could be clearly differentiated with high loading [17]. All questions showed a correlation coefficient ≥0.30, adequate for the exploratory factor analysis [17]. The cumulative variance was 60.77% based on the rotation sums of squared loadings, indicating high explanatory power [17]. The score distribution for the eight questions with verified construct validity showed that the internal consistency was Cronbach’s α = 0.9 [17].

### 2.3. Data Analysis

For statistical analyses, the SPSS version 18.0 (SPSS Inc., Chicago, IL, USA) was used. A frequency analysis was performed for the general characteristics of older adults in this study. To examine the difference in DCE competence according to various factors, a t-test and ANOVA were performed. To examine the relationship between the DCE competence and the continuous variables, a correlation analysis was performed. For an extensive analysis of the factors influencing the DCE competence in older adults, the multicollinearity among the significant factors in the univariate analysis was tested to perform a logistic regression analysis. The level of significance was set to <0.001.

## 3. Results

### 3.1. Sociodemographic Characteristics

Table 1 frequently analyzed the general characteristics of 10,073 senior citizens who completed the survey (Table 1).

### 3.2. DCE Competence Based on Sociodemographic Factors

The DCE competence significantly varied according to age: 3.06 ± 2.82 for those aged ≥65 and ≤74 years, 1.25 ± 2.07 for those aged ≥75 and ≤84 years, and 0.48 ± 1.36 for those aged ≥85 years (*p* < 001). The DCE competence also significantly differed between sex: 2.84 ± 2.85 for males and 1.80 ± 2.45 for females (*p* < 001). For spouse, a significant difference in DCE competence was found: 2.65 ± 2.79 for those living alone and 1.53 ± 2.32 for those living with a spouse (*p* < 001). The DCE competence based on the level of education varied significantly: 0.00 ± 0.08 for illiteracy, 0.60 ± 1.20 for no schooling, 1.64 ± 2.13 for elementary school, 2.98 ± 2.72 for middle school, 3.91 ± 2.86 for high school, and 5.12 ± 2.78 for university (*p* < 001) (Table 2).

### 3.3. DCE Competence Based on Self-Rated Health and Sensory Function

The DCE competence varied significantly according to self-rated health: 4.27 ± 3.10 for Very healthy; 2.98 ± 2.87 for Fairly healthy; 2.31 ± 2.70 for Moderate healthy; 1.49 ± 2.20 for Fairly unhealthy; and 1.14 ± 2.05 for Very unhealthy (*p* < 001). For visual function, a significant difference in DCE competence was found: 2.54 ± 2.77 for No discomfort; 1.72 ± 2.42 for Slight discomfort; and 1.09±2.05 for Much discomfort (*p* < 001). For auditory function, likewise, a significant difference in DCE competence was found: 2.44 ± 2.74 for No discomfort; 1.44 ± 2.26 for Slight discomfort; and 0.54 ± 1.31 for Much discomfort (*p* < 0.001) (Table 3).

### 3.4. DCE Competence Based on Education Related Factors

The DCE competence based on the value of learning varied significantly: 3.31 ± 3.13 for Very high; 2.45 ± 2.72 for Fairly high; 1.40 ± 2.10 for Moderate; 1.04 ± 1.83 for Fairly low; and 1.28 ± 2.20 for Very low (*p* < 0.001). Depending on whether the subject was engaged in an experience of lifelong learning, the DCE competence showed a significant difference: 5.97 ± 2.12 for no lifelong learning and 2.21 ± 2.66 for current participation in lifelong learning (*p* < 0.001) (Table 4).

### 3.5. Correlation of DCE Competence with Income, Cognitive Function, and Social Networks

For the level of income, cognitive function, and social networks, a significant correlation with the DCE competence was found. The highest correlation was shown between DCE competence and cognitive function (0.438), followed by income (0.298), and social network (0.259) (*p* < 0.001) (Table 5), meaning that higher DCE ability is associated with high income, high cognitive function, and wide social networks.

### 3.6. Factors Influencing DCE Competence

To identify the factors influencing DCE competence, a regression analysis was performed. This took into account the following factors, which exhibited significant variations as independent variables: sociodemographic factor (age, sex, income, spouse, education level); social network; self-rated health; sensory function (visual function, auditory function); cognitive function; education related factor (the value of learning and the experience of lifelong learning). A linear regression analysis was performed by treating the nominal variables as dummies. TOL is all over 100 and the VIF is less than 10 and there is no problem of multicollinearity, so the regression model is suitable. During the analysis, three variables (sex, spouse, auditory function) were excluded. The explanatory power of the model based on the analyzed factors was 42.7%. Among the variables, high education level, low age, high income, high cognitive function, lifelong learning experience, wide social network, high self-assessment health, high learning value, and a spouse tend to show higher DCE competency (Table 6).

## 4. Discussion

In this study, the data of the Korean National Survey of Older Adults were used to analyze the factors associated with Data Communication Equipment (DCE) competence in older adults, in order to identify the most vulnerable group with regard to information literacy, and to provide the basic data for developing the strategies towards improvement.

As age increased, DCE competence decreased. According to the 2018 Survey on the Internet Usage, the percentage of individuals using the Internet was 88.8% for those in their 60s and 38.6% for those in their 70s and above, while the percentage of individuals using the computer was 34.1% for those in their 60s and 8.7% for those in their 70s and above. The dramatic disparity between the two percentages lent support to the results in this study. Today, the level of education amongst Koreans is relatively high on a global scale, but the level varies across generations to a considerable degree [13]. Older Korean adults lived through the time of rapid changes after the Korean War, from the early economic exponential growth to industrialization and urbanization. This means that each different generation has unique experiences and resources. These differences are presumed to have led to differences in authority, income, social status, and education level, which consequently influences DCE competence [20]. Thus, the policies to improve the information literacy in older adults should take a unique approach for each age group to reflect the variation in information literacy between the relatively younger, early-phase young-old and the late-phase old-old.

A low level of education was correlated with a low level of DCE competence, which agreed with previous studies [13,21,22]. The difference in education level is a common variable influencing Internet usage across the OECD countries [7].

Older female adults showed a lower level of DCE competence than older male adults. The difference may be because older female adults tend to have a lower average level of education, as well as a lower income than their male counterparts. Most older female adults have no work or, if they do work, they work in an environment without ready access to computers [21]. The percentage of older Korean adults who are female is high. As women exhibit a longer lifespan than men, the policies regarding information literacy must consider the factors that are associated with female older adults in order to be successful.

Older adults living without a spouse showed a lower level of DCE competence. This agreed with previous studies where, compared to older adults living with a spouse, single-household elderlies showed lower performance regarding digital accessibility, capacity, and activity [23].

A low level of income was correlated with a low level of DCE competence. Among the OECD countries, the poverty rate in older adults is the highest in South Korea (48.6%, for ≥65 years), while the net pension replacement rate approaches the lowest level [24]. To use a DCE, such as computers and smart phones, the cost of device purchase and use is connected with economic status, and older adults may show limited use of smart phones due to the inability to pay such costs [25].

With a lower health status, DCE competence also decreased. The level of information literacy and health satisfaction showed a correlation [13,26,27]. Individuals whose subjective rating of health was higher tended to be more competent with understanding the health information that was found on the Internet, and showed a tendency to seek more health information using the Internet [28].

An impairment of sensory or cognitive function led to a fall in DCE competence. The lower sensory or cognitive function due to aging was shown to reduce the utilization of digital devices [29]. According to a previous study, a negative response was given by older adults regarding how difficult it was to use a device for a long time due to visual discomfort (45.3%) and to auditory discomfort (29.3%), which indicated that older adults were experiencing difficulties [16]. The decreased cognitive function led to a lower level of DCE competence, which was in line with a previous study where 58.7% of older adults found it difficult to understand new functions upon using a digital device or service; 40–50% replied that it took a long time for them to operate the device or search for the necessary information, or that they were not familiar with the general methods of use [16]. Thus, to enhance DCE competence, it is necessary to take an approach based on multi-sensory use that allows accessible use for individuals with an age-related impairment of sensory function and on a design comprising appropriate interactive factors [30].

DCE competence was low for older adults who had given a negative response to the question regarding the value of learning. In a previous study investigating the factors that prevent digital accessibility in older adults, 55.7% gave a negative response to the question ‘Do you consider yourself too old to learn a new skill?’, indicating that they faced difficulties and consequently showed a disability regarding the use of digital devices, which lent support to the results in this study [31].

DCE competence was lower for older adults without lifelong education. A digitized society is also a learning-oriented society with an increased intensity of learning in daily life. It is thus a “society of lifelong learning” where an emphasis is placed on learning throughout one’s life [32]. What is crucial, therefore, is information literacy education for middle-aged to older adults to enhance their digital competence and resolve the intergenerational differences [33].

As the social networks narrowed, DCE competence also decreased, whereas stable social networks increased DCE competence. With stable social networks, the opportunity to engage in a variety of social activities increased, making it easier for the individuals to receive social support for DCE utilization. The motivation and goals of DCE utilization were set as the older people used DCE to maintain their social networks. Many social activities and errands are now initiated, planned and performed through or with digital technology use [34]. In a study conducted on female older adults in their 70s or above [26], the main function of the smart phone included instant messaging and photo applications. The participants were found to use those applications only for close friends and acquaintances or those they frequently met offline. Selwyn et al. [35] reported that private support from family and friends has a higher relevance to Internet use among older adults than professional support or online help. This implied that the social networks had an influence on the utilization of DCE as they provided both the context of support and the purpose.

The results so far suggest that, in older Korean adults, the most vulnerable group with regard to information literacy exhibited the following variables: high age, low education level, female, living alone, low income, reduced sensory function, reduced cognitive function, a negative value of learning, no lifelong learning, and narrow social networks, indicating a need for special interest and support.

The factors that influence DCE competence in older adults were identified as follows: age, education level, income, cognitive function, spouse, social networks, lifelong learning, and the value of learning. This can be interpreted as the older people’s DCE competence depending on personal factors, such as age, education level, cognitive level, and social relationships to share their daily lives, receive help, and provide the purpose of using DCE. Therefore, improving the DCE capacity of older people requires a threshold-lowering approach, and to provide granular eye-level education, social resources to share and help to solve DCE problems, and a lower threshold for active participation in education.

In this study, although the analyzed data had sampling adequacy, there were limitations to the data interpretation. First, as the data were obtained from the 2017 Korean National Survey of Older Adults, the results could not incorporate the changes in the recent past three years from 2018 to 2021. Second, factors such as the frequency of DCE utilization could not be examined as the secondary data analysis had a limit to the inclusion of all necessary variables. Third, as the study was cross-sectional, the cause-effect relations could not be clearly determined, with a limitation that only the correlations among the variables could be addressed.

## 5. Conclusions

This study investigated the factors that influence DCE competence in older adults. The policies regarding information literacy in older adults are becoming a critical determinant in national projects [13], and a considerable negative impact is anticipated for the older people if they are not given the opportunity to improve their information literacy. This is because DCEs can serve as tools for healthy aging. In this light, further studies should be conducted with the aim of acquiring in-depth understanding of information literacy in older adults and to establish consistent and practical education programs on information literacy.

## Figures and Tables

**Table 1:** Sociodemographic characteristics (*n* = 10,073).

Variables		*n* (%)
Age	65–74	5892 (57.2)
	75–84	3532 (34.3)
	85–	875 (8.5)
	M ± SD	74.06 ± 6.70
Sex	Male	4375 (42.5)
	Female	5924 (57.5)
Spouse	Yes	6525 (64.78)
	No	3774 (37.47)
Monthly income (10,000 won)	–30	55 (0.5)
	30–50	59 (0.6)
	50–100	179 (1.7)
	100–150	183 (1.8)
	150–200	169 (1.6)
	200–	9564 (93.7)
Education	Illiteracy	627 (6.2)
	None	1766 (17.5)
	Elementary school	3452 (34.3)
	Middle school	1711 (17.0)
	High school	1751 (17.4)
	University	766 (7.6)

**Table 2:** DCE competence based on sociodemographic factors (*n* = 10,073).

Variables		DCE Competence M ± SD	t/F
Age (year)	65–74 (*n* = 5850)	3.06 ± 2.82	778.82 **
	75–84 (*n* = 3450)	1.25 ± 2.07	
	85– (*n* = 774)	0.48 ± 1.36	
Sex	Male (*n* = 4286)	2.84 ± 2.85	328.72 **
	Female (*n* = 5788)	1.80 ± 2.45	
Spouse	Yes (*n* = 6525)	2.65 ± 2.79	457.32 **
	No (*n* = 3774)	1.53 ± 2.32	
Education level	Illiteracy (*n* = 627)	0.00 ± 0.08	835.54 **
	None (*n* = 1766)	0.60 ± 1.20	
	Elementary school (*n* = 3452)	1.64 ± 2.13	
	Middle school (*n* = 1711)	2.98 ± 2.72	
	High school (*n* = 1751)	3.91 ± 2.86	
	University (*n* = 766)	5.12 ± 2.78	

** *p* < 0.001.

**Table 3:** DCE competence based on self-rated health and sensory function (*n* = 10,073).

Variables			DCE Competence M ± SD	F (*p*)
Self-rated health		Very healthy (*n* = 218)	4.27 ± 3.10	203.59 **
		Fairly healthy (*n* = 3506)	2.98 ± 2.87	
		Moderate healthy (*n* = 2348)	2.31 ± 2.70	
		Fairly unhealthy (*n* = 3519)	1.49 ± 2.20	
		Very unhealthy (*n* = 481)	1.14 ± 2.05	
Sensory function	Visual function	No discomfort (*n* = 6664)	2.54 ± 2.77	128.81 **
		Slight discomfort (*n* = 3120)	1.72 ± 2.42	
		Much discomfort (*n* = 290)	1.09 ± 2.05	
	Auditory function	No discomfort (*n* = 8271)	2.44 ± 2.74	134.29 **
		Slight discomfort (*n* = 1616)	1.44 ± 2.26	
		Much discomfort (*n* = 186)	0.54 ± 1.31	

** *p* < 0.001.

**Table 4:** DCE competence based on education related factors (*n* = 10,073).

		DCE Competence M ± SD	t/F
The value of learning	Very high (*n* = 1088)	3.31 ± 3.13	145.49 **
	Fairly high (*n* = 6427)	2.45 ± 2.72	
	Moderate (*n* = 1624)	1.40 ± 2.10	
	Fairly low (*n* = 843)	1.04 ± 1.83	
	Very low (*n* = 91)	1.28 ± 2.20	
Experience of lifelong learning	No (*n* = 9986)	5.97 ± 2.12	173.72 **
	Yes (*n* = 87)	2.21 ± 2.66	

** *p* < 0.001.

**Table 5:** Correlation of DCE competence with income, cognitive function, and social networks (*n* = 10,073).

	DCE Competence	Income	Cognitive Function	Social Networks
DCE competence	1	0.298 **	438 **	0.259 **
Income	0.298 **	1	0.190 **	0.139 **
Cognitive function	0.438 **	0.190 **	1	0.199 **
Social network	0.259 **	0.139 **	0.199 **	1

** *p* < 0.001.

**Table 6:** Factors influencing DCE competence (*n* = 10,073).

Variables	Unstandardized Coefficient		Standardized Coefficients		*p*	TOL	VIF
	B	SE	Beta	t			
(constant)	5.828	0.357		16.325	0.0001		
Education level	0.712	0.018	0.354	38.555	0.0001	0.674	1.483
Age	−0.102	0.003	−0.248	−29.765	0.0001	0.821	1.218
Income	0.000	0.000	0.115	14.413	0.0001	0.886	1.128
Cognitive function	0.069	0.007	0.099	10.555	0.0001	0.649	1.540
Experience of lifelong learning	0.605	0.061	0.076	9.924	0.0001	0.976	1.025
Social networks	0.073	0.008	0.072	9.101	0.0001	0.897	1.114
Self-rated health	−0.182	0.022	−0.067	−8.280	0.0001	0.867	1.154
The value of learning	−0.201	0.027	−0.059	−7.495	0.0001	0.913	1.095
Spouse	0.133	0.034	0.031	3.934	0.0001	0.928	1.078
Visual function	−0.101	0.039	−0.020	−2.599	0.009	0.939	1.065
F (*p*)	688.172 (*p* < 0.001)						
Adjust R^2^	0.427						

TOL, tolerance; VIF, variance inflation factor.

## Data Availability

The datasets analyzed during the current study can be accessed from the Korean public data portal. https://www.data.go.kr/data/15004296/fileData.do (accessed on 30 December 2021).
